# Fungicide effects on human fungal pathogens: Cross-resistance to medical drugs and beyond

**DOI:** 10.1371/journal.ppat.1010073

**Published:** 2021-12-09

**Authors:** Rafael W. Bastos, Luana Rossato, Gustavo H. Goldman, Daniel A. Santos

**Affiliations:** 1 Faculdade de Ciências Farmacêuticas de Ribeirão Preto, Universidade de São Paulo, Ribeirão Preto-SP, Brazil; 2 Federal University of Grande Dourados, Dourados-MS, Brazil; 3 Laboratory of Mycology, Federal University of Minas Gerais, Belo Horizonte-MG, Brazil; Rutgers University, UNITED STATES

## Abstract

Fungal infections are underestimated threats that affect over 1 billion people, and *Candida* spp., *Cryptococcus* spp., and *Aspergillus* spp. are the 3 most fatal fungi. The treatment of these infections is performed with a limited arsenal of antifungal drugs, and the class of the azoles is the most used. Although these drugs present low toxicity for the host, there is an emergence of therapeutic failure due to azole resistance. Drug resistance normally develops in patients undergoing azole long-term therapy, when the fungus in contact with the drug can adapt and survive. Conversely, several reports have been showing that resistant isolates are also recovered from patients with no prior history of azole therapy, suggesting that other routes might be driving antifungal resistance. Intriguingly, antifungal resistance also happens in the environment since resistant strains have been isolated from plant materials, soil, decomposing matter, and compost, where important human fungal pathogens live. As the resistant fungi can be isolated from the environment, in places where agrochemicals are extensively used in agriculture and wood industry, the hypothesis that fungicides could be driving and selecting resistance mechanism in nature, before the contact of the fungus with the host, has gained more attention. The effects of fungicide exposure on fungal resistance have been extensively studied in *Aspergillus fumigatus* and less investigated in other human fungal pathogens. Here, we discuss not only classic and recent studies showing that environmental azole exposure selects cross-resistance to medical azoles in *A*. *fumigatus*, but also how this phenomenon affects *Candida* and *Cryptococcus*, other 2 important human fungal pathogens found in the environment. We also examine data showing that fungicide exposure can select relevant changes in the morphophysiology and virulence of those pathogens, suggesting that its effect goes beyond the cross-resistance.

## 1. Introduction

*Candida* spp., *Cryptococcus* spp., and *Aspergillus* spp. are among the 3 most lethal human pathogenic fungi [1] as they can cause severe systemic infections, which may be fatal even when treated [2]. The treatment relies on a limited arsenal of antifungal drugs from 3 classes: polyenes, echinocandins, and azoles [2,3]. The main antifungal effect of polyenes (for example, amphotericin B) is through binding to a conserved ergosterol region forming large extramembraneous aggregates that remove ergosterol from lipid bilayers [4,5], while echinocandins (caspofungin, anidulafungin, micafungin, and, more recently, rezafungin) disrupt the cell wall as they inhibit noncompetitively the 1,3-D-glucan synthase, an important enzyme for cell wall biosynthesis. Azoles, which are classified as imidazoles (ketoconazole and miconazole), and triazoles (fluconazole, itraconazole, voriconazole, posaconazole, and isavuconazole), interrupt ergosterol synthesis by inhibiting lanoterol-14α-D-demethylase encoded by the orthologous *ERG11* (in yeasts) and *cyp51* (in *Aspergillus fumigatus*) [2,6,7]. This prevents the conversion of lanosterol into 4,4-dimethyl-8,14,24-trienol, reduces the ergosterol levels on the cell membrane, and accumulates toxic sterols, affecting the membrane integrity and permeability, ultimately inhibiting fungal growth [6–8].

One of the reasons for treatment failure and the high number of deaths caused by systemic mycoses is the emergence of resistance [9–11]. Microbiological resistance is defined as the inability of an antifungal to kill or inhibit the fungal growth in vitro [6,12,13] and can be divided into 2 classes: (i) primary or intrinsic resistance, when a microorganism is naturally resistant to a drug, without previous exposure; and (ii) secondary resistance, when resistance mutations evolve in the population and are selected upon exposure to an antifungal [6].

Several cases of isolation of azole-resistant strains from patients with no prior antifungal therapy have been reported, suggesting that other routes might be driving antifungal resistance [14–18]. Intriguingly, antifungal resistance also happens in the environment since resistant strains have been isolated from plant material, soil, decomposing matter, and compost [19–27]. This fact raises an important question: How does resistance to azoles arise in environmental isolates?

One answer to this question is based on the massive use of fungicides during preharvest in grain- and grass-growing environments and postharvest to prevent spoilage [26,28]. In addition, azoles are used for preserving paintings, coatings, and wallpaper pastes and are typically applied to mattresses to avoid fungal growth [26]. Environmental triazoles also share the same mechanism of action as medical triazoles and have been extensively used for controlling fungal phytopathogens [29,30]. Because of that, and since certain potential human pathogens can be easily isolated from plant material and soil, the most accepted hypothesis is that agrochemicals, especially 14α-demethylase inhibitors (DMIs), operate as a selection pressure for the emergence of resistant strains in the environment (fungicide-driven drug resistance route) [26,31]. Based on that, this review discusses classic and recent studies showing that environmental azole exposure selects cross-resistance to medical azoles in *A*. *fumigatus*, with a focus on the mechanisms involved. In addition, we also discuss how this phenomenon can affect *Candida* and *Cryptococcus*, other 2 important human fungal pathogens found in the environment.

## 2. *Aspergillus fumigatus*

### 2.1 Habitat, clinical manifestations, treatment, and resistance prevalence

*Aspergillus fumigatus* is a saprophytic fungus found in soil, crops, seeds, air, leaves, flowers, and indoor environments [15,17,19–21,26,32–36]. It also causes a wide range of chronic and life-threatening infections, such as allergic bronchopulmonary aspergillosis (ABPA), chronic pulmonary aspergillosis (CPA), and invasive pulmonary aspergillosis (IPA) [37]. Such diseases are treated with a restricted arsenal of antifungals from 3 classes: azoles, polyenes, and echinocandins [37–39]. Specifically, the triazoles (voriconazole, itraconazole, posaconazole, and isavulconazole) are the most indicated as the first-line therapy [38,40] and liposomal amphotericin B (polyene) and echinocandins as second-line choices [38,40,41]. Unlike echinocandins and polyenes, resistance to azoles is relatively common and has been increasing since the first *A*. *fumigatus* azole-resistant strains were reported in 1997 [42].

The incidence of clinical *A*. *fumigatus* triazole resistance varies according to the country and the patient from which it is isolated. In European countries, clinical resistance ranges from 0.6% to 30%, having reached the highest rate (>20%) in the Netherlands, United Kingdom, and Germany [43,44]. Outside Europe, azole resistance has been detected in China (5.5%), India (1.7%), Iran (3.5%), Japan (12.7%), Thailand (3.2%), Australia (2.6%), and the United States (0.6% to 11.8%) [15,32,43,45–48]. In South America, Brazil, Peru, Mexico, and Argentina have also reported triazole-resistant isolates [24,49–53]. The clinical implications of an infection caused by an antifungal-resistant strain are not totally revealed and not always related to therapeutic failure [43]. Nonetheless, some studies have shown that resistance may ultimately lead to a poor outcome [9–11,54].

Triazoles are not mutagenic compounds, which means that resistance occurs when genetic changes in the progeny of *A*. *fumigatus* are selected during reproduction. In *A*. *fumigatus*, 3 modes of reproduction can happen: asexual, sexual, and parasexual. Through asexual sporulation, common in nature, *A*. *fumigatus* produces an abundant number of spores (conidia). Even though the progeny from asexual reproduction is clonal, many conidia may harbor spontaneous mutations, ensuring genetic diversity. If one or more mutations give the conidia a better ability to survive and grow under certain stresses (for example, triazole exposure), the mutant will proliferate and might surpass the growth of the wild-type spore. This selective pressure can happen in any environment containing azoles [55,56].

Although many studies have proved that azole therapy can drive inpatient resistance to emerge in *Aspergillus* spp. clones [57–66], this route does not explain all cases observed in the genus. Actually, it is estimated that only one-third of the resistant strains arise from in-host adaptation, remarkably those suffering from aspergilloma, allergic or chronic aspergillosis, and predisposing conditions as lung cavities or cystic fibrosis (CF) [11,64]. The main evidence indicating another route is the azole-resistant *A*. *fumigatus* isolated from azole-naive patients, which accounts for 64% to 71% of the multiresistant *A*. *fumigatus* isolates [16,67,68]. Mellado and colleagues recovered 13 multiple triazole-resistant *A*. *fumigatus* strains from patients at different hospitals in the Netherlands—4 of them from individuals with no history of azole treatment [16]. In those cases, the isolates were not only resistant to itraconazole but also had high MIC values of voriconazole, posaconazole, and ravuconazole [16,69]. Subsequently, many studies in different countries have also identified azole-resistant isolates from patients not previously treated with these drugs [46,68,70,71].

Two main hypotheses have been raised to explain this phenomenon: (i) person-to-person transmission of resistant strains; or (ii) infection by an isolate that acquired the resistance mechanism in the environment [26,30]. The first hypothesis has little scientific support because person-to-person or person-to-environment transmissibility has been considered rare or inexistent. In the past, it was thought that transmission happens only through direct donor-to-recipient contact and infected wounds, as most of the transmission happens via aerosolized spores [30]. However, Engel and colleagues proved that *A*. *fumigatus* could be recovered from cough aerosols from CF patients [72], thus opening the possibility of patient-to-patient and patient-to-environment transmission. Further experiments, however, are still necessary to better detail the transmission of *A*. *fumigatus* by coughing. Nevertheless, aerosolized *A*. *fumigatus* conidia from patients could not explain all the resistance found in azole-naive patients due to its frequency, and the aerosolized conidia from environmental sources seem to represent a vaster and more constant source of infection [72].

### 2.2 Fungicide-driven resistance: Epidemiological, experimental, and field data

**Fig 1:**
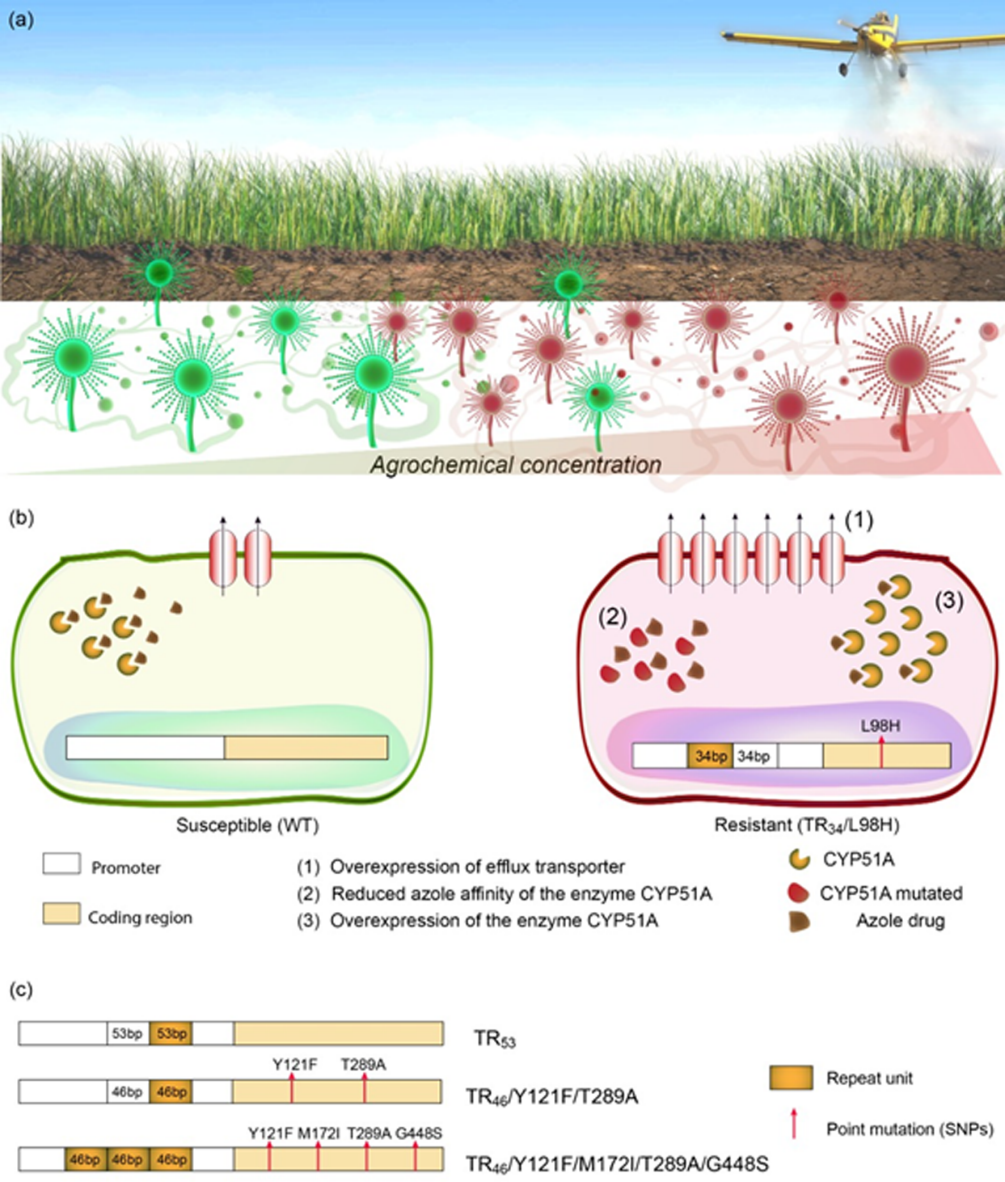
Fungicide exposure effects on *Aspergillus fumigatus*. (a) Azole-susceptible and azole-resistant *A*. *fumigatus* can be identified in both fungicide-free and fungicide-containing soils and plant-based materials. There is an enrichment, however, of azole-resistant *A*. *fumigatus* in niches containing fungicides. (b) Azole-resistant *A*. *fumigatus* isolated from places holding fungicides may present some alterations compared to susceptible isolates that confer them cross-resistance with medical azoles, such as overexpression of efflux pumps and the azole-target enzyme, CYP51A, and CYP51A with a reduced azole affinity. The last 2 physiological changes are due to mutations in the gene *cyp51A*. The most common mutations are a pair of 34-bp sequence (in tandem) in the gene promoter (TR_34_), which lead to overexpression of *cyp51A*, together with a mutation that results in leucine replacement by histidine at position 98 (L98H) in the enzyme CYP51A, reducing the affinity of the enzyme to the azole drugs. (c) Other tandem repeat mutations combined or not with point mutations in the gene *cyp51A* conferring cross-resistance between environmental and medical azoles also can be detected in azole-resistant *A*. *fumigatus* isolated from fungicides-containing places. It is important to notice that the alterations represented correspond to amino acids and not in the DNA and that other tandem repeat mutations have already been observed in the clinical sets, but only TR_34_, TR_46_, and TR_53_ have been describing in environmental strains.

Many epidemiological and experimental data corroborate the theory that the DMIs used in the wood and textile industries, and especially those employed in agriculture, may select azole resistance in *A*. *fumigatus* in the environment [29,33,46,73,74] (Fig 1A). These studies presenting data supporting fungicide-driven resistance can be categorized into 4 groups: (i) those in which resistant strains were found in both patients and environment [19–24,32–34,49,68,73–81]; (ii) studies attesting cross-resistance between environmental and medical azoles in isolates from both sources [20,22,30,33,46,75,82]; (iii) investigations demonstrating that susceptible isolates could become resistant when exposed to environmental azoles [29,74,83–85]; and (iv) those proving that more resistant strains could be recovered from places or periods at which the fungicides were applied [20,86].

Classically, the studies in the Netherlands started to shed light on how environmental azole exposure could lead to cross-resistance to medical azoles [26,30]. First, they demonstrated that itraconazole-resistant *A*. *fumigatus* could be isolated from indoor environments (including patient rooms at hospitals), as well as from cultivable soils, seeds, leaves, and compost—but never from azole-naive soils. These resistant strains also posed high resistance to 2 fungicides, metconazole and tebuconazole, thus demonstrating cross-resistance between medical and environmental azoles [26]. Interestingly, 13 out of the 15 resistant strains isolated from the environment had the same mutation in the gene that encodes the azole-target enzyme (*cyp51A*) [26], which was identical to the isolate identified in the clinical isolates [14]. Such mutations led to a leucine replaced by histidine at position 98 (L98H) in the enzyme CYP51A, along with a pair of 34-base pair (bp) sequence (in tandem) in the gene promoter region (TR_34_) (TR_34_/L98H) [16]. The 34-bp sequence in tandem in the *cyp51A* promoter induces overexpression of *cyp51A* (about 8-fold) [16], and the point mutation hinders the interaction between the drug and the target enzyme [30] (Fig 1B). This combination of mechanisms results in a consistent itraconazole resistance and variable voriconazole, posaconazole, and isavuconazole susceptibility [30,34,68]. Frequently, TR_34_/L98H also confers a pan-azole resistance, both to medical and environmental azoles [26,30]. Coincidentally, the first resistant clinical isolate carrying TR_34_/L98H was reported infecting a patient in 1998 [14,30], just a few years after triazole fungicides had been introduced into the Netherlands [30], which suggests that this mutant could had emerged after azole fungicide contact in the field. Eventually, the TR_34_/L98H mutation was identified in many other European countries, and also in Asia, North and South America, Australia, and Africa [25,43].

The tandem repeat mutation was also identified in DMI-resistant phytopathogens [82,87], strongly suggesting that this is a common resistance mechanism among molds exposed to these fungicides. *Penicillium digitatum*, for example, contains tandem repeat mutations varying from 126 bp to 199 bp, which have been associated with DMI resistance [88,89]. However, other resistant isolates of plant pathogens, such as *Pyrenopeziza brassicae*, *Monilinia fructicola*, and *Venturia inaequalis*, have fragments inserted in *cyp51A* promotor (fragments from 65 bp to 553 bp) [90–92] instead of tandem repeat alteration. In general, both genetic variations result in overexpression of *cyp51A* as in *A*. *fumigatus* [82,87].

If DMIs are really the stressors leading to selection of these mutations in the environment, they should probably share similar molecular structures to clinical azoles and dock similarly to them at the azole-target enzyme in *A*. *fumigatus*. In order to address these questions, Snelders and colleagues carried out molecule alignment and docking studies using homology modeling of *cyp51A*. They identified 5 DMIs, propiconazole, bromuconazole, tebuconazole, epoxiconazole, and difenoconazole, which share structural molecular characteristics to medical triazoles, suggesting that they could select cross-resistance in *A*. *fumigatus*. These DMIs also assume a similar configuration when docking to the target enzyme and act against wild-type but not against multi-triazole-resistant *A*. *fumigatus* [30], further supporting the idea of DMI as a selection pressure.

Other resistance mechanisms involving promotor duplications, either combined or not with single nucleotide polymorphisms (SNPs), have been described in clinical and environmental strains (Fig 1C). TR_53_ (2 copies of a 53-bp sequence in tandem in *cyp51A*) was the second mechanism discovered [30] and thought to be restricted to clinical isolates until it was identified in resistant *A*. *fumigatus* strains isolated from flower fields in Colombia [49]. TR_46_/Y121F/T289A (with 2 copies of a 46-bp sequence in tandem in *cyp51A*, combined with 2 SNPs) (Fig 1C) was also identified in both clinical and environmental isolates [20,48,49,51,71,73,74,93,94]. This mutation provides resistance especially to voriconazole and in some cases to other medical azoles and environmental fungicides [73]. TR_46_/Y121F/T289A was first reported in the Netherlands [93] and subsequently in Belgium [95], India [73], Denmark [71], Germany [96], Colombia [24,49], and China [86]. The spreading of TR_46_/Y121F/T289A is worrisome, as it can cause high resistance to voriconazole, which is recommended as the first-line therapy for many aspergillosis [97].

Recently, another promoter-repeat mutation (a triple 46-bp promoter repeat), combined with 4 SNPs (TR463/Y121F/M172I/T289A/G448S), which leads to a pan-triazole resistance, was discovered (Fig 1C) [20]. The isolates harboring these mutations came from compost heaps containing azole fungicides and *A*. *fumigatus* clinical isolates from the Netherlands [20]. Moreover, additional tandem repeats in *cyp51A* gene, either combined or not with SNPs, were reported in environmental azole-resistant strains, such as TR464/Y121F/M172I/T289A/G448S [20], TR34/L98H/S297T/F495I [22,86], TR46/Y121F/M172I/T289A/G448S [19], TR92/Y121F/M172I/ T289A/G448S [19], and point mutations without tandem repeat alterations, for example, P216L [33], A284T, G448S, P222Q [74], G54R [34], G138S, Y433N, and N248K [85].

In the environment, azole-resistant isolates harboring the aforementioned genetic modifications have been isolated from several places and materials, including leaves, plant seeds, soil samples, flowerbeds, compost, hospital surroundings, and air samples [19,20,22,24,26,34,49,93,98]. In this way, some researchers have been reporting potential hotspot to isolate those mutants (especially TR34/L98H and TR46/Y121F/T289A), including soils from strawberry fields in China [22]; azole-exposed compost [20], flower bulb waste, green waste material, and wood chippings in the Netherlands [19]. These environments contain several characteristics that may facilitate not only the emergence of azole-resistant strains, but also their maintenance, and spread [19,20]. Such chacharacteristic are beyond the scopus of this review and has been recentely well discussed by Burks and colleagues [98]. Besides the fact that not all the soil or culture seems to be favorable for the emergence of resistant strains, it appears that are some DMIs more prone to select mutations in *A*. *fumigatus* and cause cross-resistance with medical azoles, such as propiconazole, bromuconazole, tebuconazole, epoxiconazole, difenoconazole, prothioconazole, and azaconazole [19,30].

Mutations in the *cyp51A* promoter and its open reading frame (ORF) causing overexpression and/or significant changes in the conformation of lanosterol 14α-demethylase are the primary azole resistance mechanisms in clinical and environmental *A*. *fumigatus* isolates. However, azole-resistant strains with wild-type *cyp51A* have been found, suggesting other resistance means unrelated to *cyp51A* modifications [29,81,86]. Cui and colleagues exposed azole-susceptible strains to liquid culture medium and soil treated with tebuconazole and then recovered 12 resistant isolates without any alteration in the *cyp51A* gene [29]. The mRNA quantitative analysis showed that some of these isolates overexpressed the genes encoding a transcription factor involved in resistance (*AtrF*), 2 efflux pumps (*AfuMDR1*, *AfuMDR2*), and paralogue genes for the azole-target enzyme (*cyp51A* and *cyp51B*) [29]. Another study also demonstrated that the fungicide propiconazole could select resistance by causing overexpression of *cyp51A* and the efflux pump genes *AfuMDR3* and *AfuMDR4* [85]. Overall, these data show how diverse the mechanism behind azole resistance in *A*. *fumigatus* is (Fig 1B) and that researchers should also look for alterations beyond the *cyp51A* gene.

The role of asexual reproduction and in vitro and in vivo resistance acquisition in *A*. *fumigatus* is already well defined and discussed in this paper. In contrast, the importance of sexual and parasexual cycles are not totally revealed. There is building evidence showing that sexual cycle of *A*. *fumigatus* plays a vital part in its resistance development, thus accounting for the genetic diversity. In this sense, Camps and colleagues verified that TR_34_/L98H strains could outcross with wild-type isolates with diverse genetic backgrounds [99], and Zhang and colleagues obtained TR_46_^3^ mutation outcrossing 2 TR_46_ strains that were isolated from the same azole-containing compost, possibly through unequal crossing over between the double tandem repeats (TRs) during meiosis [20]. Sexual reproduction, which requires 2 different mating types, results in new genotypes, which may be a source of diversity within azole-resistant isolates in vitro [86]. In turn, the parasexual cycle, performed through the hyphal plasmogamy, nuclear exchange and fusion, and subsequent haploidization, plays a role in azole resistance development in diploid *A*. *fumigatus* isolated from CF patients [100]. Nevertheless, its function in environmental resistance acquisition is still unknown.

## 3. The other side of the story

The hypothesis that DMI could be prompting resistance in *A*. *fumigatus* is not unanimously accepted. Hollomon, for instance, stated that it was unlikely that selection for resistance occurred in soil [28]. He verified that the levels of fungicides available at the upper 10 cm of soil were very low (maximum exposure concentrations (MECs), between 0.3 and 0.4 mg/kg), especially when compared to the exposure concentrations of triazole drugs in patients (approximately 11 mg/L of blood serum) [28]. Indeed, some studies have proved that higher concentrations of fungicides are required to obtain resistant isolates from azole-contaminated soils (1.0 to 10.0 mg/kg of propiconazole and 0.5 to 5.0 mg/kg of tebuconazole) [29,85]. Nevertheless, in his critical analysis, Hollomon considered the results from a single-spray application [28]. In turn, other authors demonstrated that, for example, the level of propiconazole deposited in the soil was approximately 0.5 to 2.0 mg/kg when it was sprayed on plants 2 to 3 times, with an interval of 7 to 10 days, which is the recommended application regimen for this DMI [101]. Therefore, it is plausible to imagine that the residual DMI in the soil might be enough to select resistant isolates.

Another critical point raised by Hollomon was the lack of experimental data detecting any preexisting resistant isolates in the cultivable fields and showing how their frequency rose after the azole spraying [28]. Recently, Barber and colleagues conducted a systematic study, in which they sampled 10 agricultural sites in Germany over 3 years [102]. In their research, they consider both conventionally managed fields, where azole fungicides were applied, and those in organic farming systems, which did not use these compounds. Although they were able to isolate azole-resistant strains carrying the most common mutations, the results exhibited only a modest decrease in azole susceptibility after the growing season and azole exposure [102]. Hence, this study did not prove a direct and incontestable link between azole application in the field and increased azole resistance in *A*. *fumigatus*. Other studies have also failed in connecting fungicide usage and *A*. *fumgiatus* increasing resistance. van der Torre and colleagues recovered over 86 *A*. *fumigatus* from soil-covered root vegetables and other fresh produce in the UK, and none was azole resistant [103,104]. Similarly, Astvad did not detect resistance from any of the 113 isolates from soil in Denmark. Additionally, no pan-azole-resistant mutant (TR_34_ or TR_46_) was found from 180 strains isolated from soil samples in UK (90 from untreated wheat crops and 90 from plots sprayed with foliar fungicides), neither other 30 strains isolated from permanent grass land [104].

On the other hand, some authors showed consistent data attesting that azole-resistant isolates are significantly more common in DMI-containing places, such as sawmills that use fungicides to preserve wood compared to the ones that do not [33], soils from azole-treated agricultural sites versus urban areas [23], and compost heaps containing azoles in relation to azole-free ones [20]. Furthermore, Cao and colleagues, in a comprehensive study aiming to isolate resistant *A*. *fumigatus* from paddy soils, found that the prevalence of azole-resistant isolates is positively correlated with the residual levels of azole fungicides in the soil [86] (Fig 1A).

Overall, these data indicate that the DMI used in the agriculture and wood industry could be the main responsible for selecting resistant strains of *A*. *fumigatus*. Nonetheless, this process depends on some factors, such as the amount of azole applied and remaining in the environment (residual azole), the frequency of application, the type of azole employed, whether the azoles are used in a mixture or as an individual drug, and the interval between applications [29,33,86].

### 3.1 Fungicide effects on morphology, physiology, and virulence: What we know and it is missing?

Other aspects of *A*. *fumigatus* exposure to fungicides have been scarcely studied, such as its effect on virulence, metabolism, morphology, and fitness cost. Resistance mutations usually happen at a cost, as in the absence of an antifungal drug, the resistant genotype is less fit than the wild-type isolates [56]. Consequently, the mutant can disappear in the drug-free environment or become less virulent due to the fitness cost. Faria-Ramos reported that prochloraz-adapted colonies of *A*. *fumigatus* macroscopically became mostly white, losing the typical pigmentation due to nonconidiation, which must affect spreading and infectiveness [83,105]. In contrast, strains carrying *cyp51A* mutations, as TR_34_/L98H and TR_46_/Y121F/T289A, apparently do not have any fitness cost, as they are found dispersed worldwide in both azole-containing and azole-naive environments, coexisting with wild-type strains [105,106].

Nonetheless, little is known about the apparent absence of fitness cost in these and other fungicide-exposed mutants. Some hypotheses that still need scientific proof have been raised as follows: (i) resistant strains exhibit fitness cost in some particular environments, and the strains have only been tested under optimal laboratory conditions; (ii), TR_34_/L98H and TR_46_/Y121F/T289A could have developed a compensatory evolutionary mechanism, meaning that mutations might have counterbalanced any fitness cost by exposition to an azole-free environment; and (iii) tandem repetitions in the promoter could have been the compensatory mutation for the point mutations in *cyp51A* [56].

In summary, recent data have filled some gaps and reinforced the theory of fungicide-driven azole resistance in *A*. *fumigatus*. However, future research should also consider *cyp51A*-independent mechanisms and other fungal aspects (fitness cost, virulence, and metabolism) of azole resistance development.

## 4. *Candida* spp.

### 4.1 Habitat, clinical manifestations, treatment, and resistance incidence

*Candida* is a medically important polyphyletic fungal genus with more than 300 different species, of which 20 are potentially pathogenic to humans and other mammals [107,108]. *Candida albicans*, *Candida glabrata*, *Candida parapsilosis*, and *Candida*. *tropicalis* are part of human microbiota responsible for most of infections involving this fungus [109–113]. These infections, collectively called candidiasis, range from superficial mycoses and deep-seated (intra-abdominal abscesses, peritonitis, and osteomyelitis) to invasive infections (candidemia) [110,114].

Candidiasis can be treated with polyenes, echinocandins, and, especially, azoles [115]. However, the azole therapy has been presenting an increasing limitation due the number of clinical azole-resistant strains that have been isolated lately, especially among the non-*albicans Candida* species [111,116–118]. This can be linked to the massive use of fluconazole as prophylaxis (in patients considered at risk of infection) that could be selecting secondary resistance [119–122]. Intriguingly, although in-host resistance acquisition is the main route of azole resistance development in *Candida* spp., the isolation of azole-resistant strains from patients with no prior history of antifungal treatment has become common [123–128]. One of the explanations for this phenomenon may be in the environment [129].

Although the environment is not the primary reservoir for most of the *Candida* spp., they are also found in soils, trees, fruits, and water [129–132]. Indeed, it seems that some species are more related to specific niches, as *C*. *tropicalis* in soils, while others, such as *C*. *albicans*, can be found in multiple niches (fruits, soil, and plant matter) [129].

### 4.2 Fungicide-driven resistance: Epidemiological, experimental, and field data

Similar to *A*. *fumigatus*, *Candida* isolates from the environment may present reduced susceptibility or resistance to clinical azoles [129,131]. This fact raises the question if any environmental factors exist acting as a selecting pressure and affecting the fungus before contact with the host. Considering that *Candida* spp. is found in the environment and may acquire resistance in that place, the hypothesis that fungicides, especially environmental azoles, could be the stressor-selecting pressure has gained more attention.

Some observations support the link between the agricultural use of azole agrochemicals and the emergence of *Candida* spp. resistance [133]. First, it has been shown that the fluconazole MIC values are higher in *Candida* isolated from the surface of nonorganic fruits (sprayed with fungicides) compared to those collected from organic ones (without agrochemical) (16 to 64 g/L versus 1 to 8 mg/L) [134]. Secondly, *C*. *tropicalis* from the soil of Taiwan had a reduction in fluconazole susceptibility and showed genetical relatedness with clinical and less azole-susceptible strains. In addition, these isolates were more resistant to agricultural azoles, suggesting a cross-resistance between environmental and clinical azoles [131]. The cross-resistance between these substances has been also shown in *C*. *albicans* obtained from the oropharynx of HIV–positive people, which had resistance to fluconazole and high MIC to agricultural azoles (fluquinconazole, penconazole, tebuconazole, and triadimenol) [135].

Obtention of cross-resistance can also be achieved in vitro to exposing yeasts to agricultural azoles. Fluconazole and posaconazole resistance, for example, were selected in *C*. *glabrata* after a previous exposure to the fungicide prochloraz [136]. In addition, susceptible *C*. *parapsilosis* species complex became more resistant to fluconazole, itraconazole, and voriconazole after being cultured in a medium supplemented with the fungicides tetraconazole and tebuconazle, similarly as happened in the positive control using fluconazole [137,138].

The idea that fungicide-driven resistance in human pathogens has also been used to explain the origin of new multidrug resistance in *Candida* species, such as *Candida auris* [139,140]. *C*. *auris* is an emerging yeast, frequently resistant to fluconazole, and recently reported in clinical settings worldwide that may have its origin in the environment [141]. This hypothesis is supported by the new study of Arora and colleagues, who, for the first time, isolated this species from the environment. *C*. *auris* was found in salt marsh virgin habitats (areas with no human activity) and sandy beaches, which suggests that prior to its recognition as a human pathogen, it existed as an environmental fungus [141]. One isolate demonstrated to be less antifungal resistant, which could reinforce the hypothesis that drug resistance in clinical strains isolated in other parts of the world emerged from induction by fungicides [130]. However, so far, it is not known if *C*. *auris* lives in cultivable soils or in plant materials, where they could be in contact with fungicides. Even though, due its multidrug resistance, it has been proposed that agrochemical exposure may be related to the *C*. *auris* resistance [139,140]. In fact, distribution maps of azole fungicides usage within the US matched the reported scattering of *C*. *auris* [142]. More experiments and field data are necessary to test such hypothesis.

**Fig 2:**
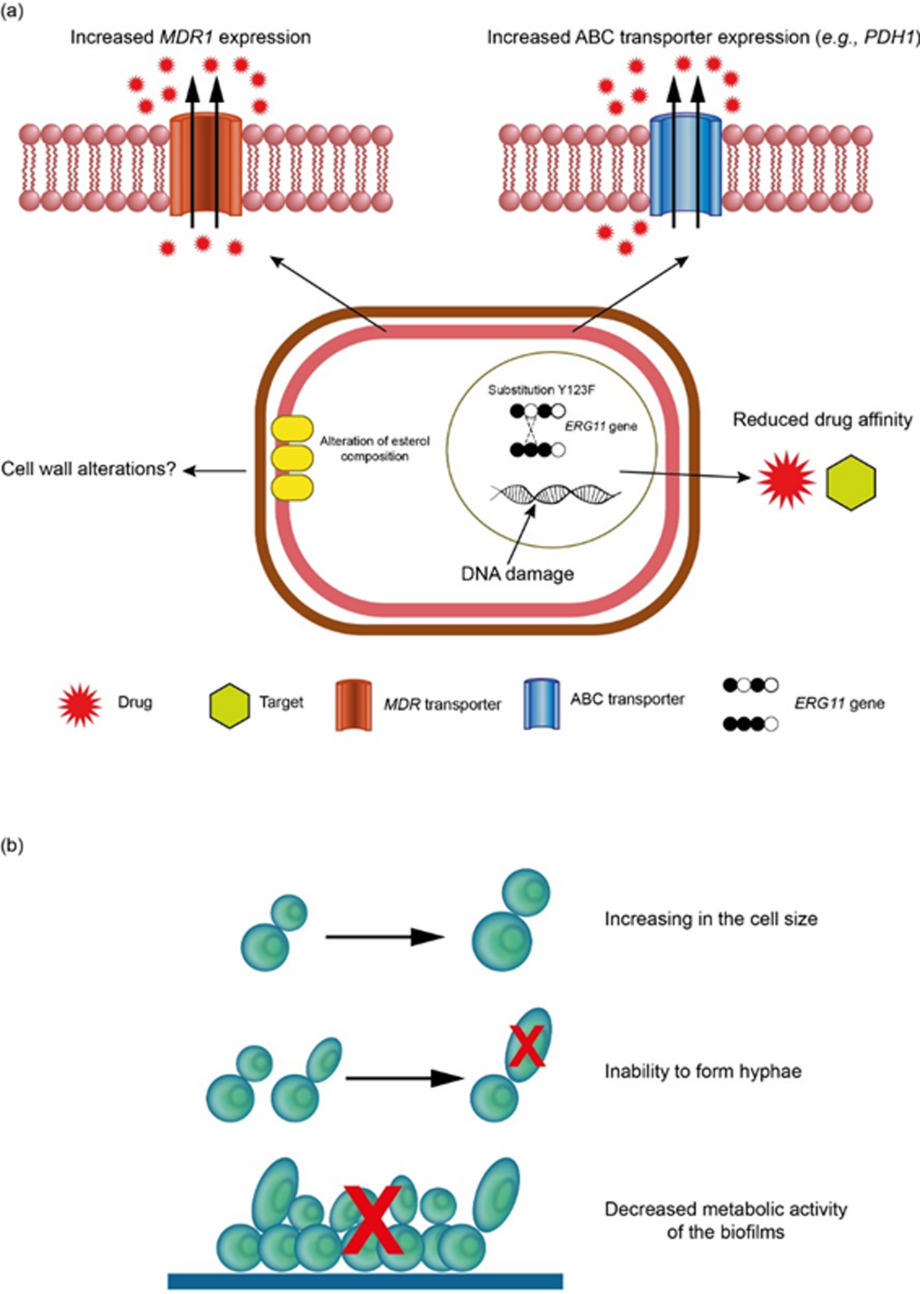
Cellular alterations induced by fungicides exposure in *Candida* spp. (a) Mechanisms of resistance induced by fungicides in *Candida* spp. Azole resistance triggered by fungicide exposure shows up-regulation of ABC multidrug transporters, such as *PDH1*. In addition, amino acid substitution Y132F in the *erg11* gene can occur, suggesting that this selected resistance is mainly associated with increased drug efflux through ATP-dependent pumps. Sterol composition and DNA damage are also consequences of fungicide exposure. (b) Alterations in morphophysiology and virulence of *Candida* spp. caused by fungicides. *Candida* spp. exposure to fungicides showed an expanded cell size, inability to form hyphae, and significantly altered time of adhesion and decreased the metabolic activity of biofilms. ABC, ATP-binding cassette.

Primary and secondary azole resistance mechanisms are well studied and understood in *Candida* spp. Several mechanisms have been described, being the most important the overexpression of *ERG11* and efflux pumps (*MDR*, *CDRs*) genes and alterations in ERG11p [111]. Coincidently, agricultural azoles select cross-resistance by using the exact mechanisms underlying fluconazole resistance (Fig 2A) [137,138]. Prochloraz induces the up-regulation of the *ATP binding cassette* multidrug transporter genes (*PDH1*) and the transcription factor that may regulate them (*PDR1*) but seems to not select any important mutation in *ERG11* [136]. Alike, Rocha and colleagues demonstrated that *C*. *parapsilosis* exposed to tetraconazole and with cross-resistance to clinical azoles increased drug efflux through pumps, such as MDR1p and CDRp [143] (Fig 2A). Lately, Brilhante and colleagues showed that tebuconazole- and tetraconazole-exposed *C*. *parapsilosis* species complex strains had cross-resistance due to overexpression of *ERG11* but not of efflux pump genes [137]. Also, sterol composition in *C*. *parapsilosis* (sensu stricto) and *Candida orthopsilosis* tend to be different after fungicide exposure [137], what may be related to azole resistance if it supports the membrane integrity. Altogether, these data show the diverse azole mechanisms that can be selected by fungicides (Fig 2A).

### 4.3 Fungicide effects on morphology, physiology, and virulence: What we know and it is missing?

In addition to cross-resistance, agrochemicals can affect the morphophysiology and virulence of *Candida* spp. (Fig 2B). Tebuconazole altered the metabolism of *C*. *parapsilosis* (sensu stricto) at the time of adhesion and decreased the metabolic activity of biofilms [137]. Species of azole-tolerant biofilm-producing non-wild-type *C*. *albicans* were found colonizing agricultural soils cultivated with azole fungicides [144]. The influence of fungicides on the development phases of *Candida* spp. may mimic the state of an in vivo infection of yeast colonies occurring in a natural environment. Specifically, *C*. *albicans* and *Candida pulcherrima* showed an expanded cell size after exposure to different concentrations of Tango Star (epoxiconazole and fenpropimorph), and *C*. *albicans* was not able to form hyphae (Fig 2B). Tango Star, which inhibits ergosterol synthesis, may contribute to depleting the intracellular pool of ergosterol while blocking the transition of blastospores during hyphae formation [145]. The overall response to agrochemical stress in *C*. *glabrata* and to a lesser extent in *C*. *tropicalis* was the selection of subpopulations with increased fatty acid unsaturation rates [145]. Treatments with Tango Star also aggravated the total DNA damage in *C*. *pulcherrima* cells (Fig 2B) [146].

In summary, there is some field and experimental data demonstrating that fungicides may be inducing resistance to clinical azoles in *Candida* spp. mainly through activation of overexpression of efflux pumps and *ERG11* genes. They are also affecting its morphophysiology; however, it is unclear, if those alterations impact *Candida* virulence. Based on that, the use of azoles in human medicine and the environment requires surveillance and restrictions to minimize the risk of selecting azole resistance in *Candida*.

## 5. *Cryptococcus* spp.

### 5.1 Habitat, clinical manifestations, treatment, and resistance incidence

*Cryptococcus neoformans* and *Cryptococcus gattii* (also called *C*. *neoformans* and *C*. *gattii* complex) are encapsulated basidiomycetous yeasts and the most medically relevant species within the genus *Cryptococcus*, causing infections called cryptococcosis [147]. Although *C*. *neoformans* has been typically found in association with birds, isolated from their nests and excrements [147–149], both species live predominately in niches related to plant material, such as bark and trunk cavities of trees, fruits, underlaying soil, and decaying wood. They can be isolated from trees of *Eucalyptus* spp. (eucalyptus), *Olea* (olive trees), *Ceratonia* (carob trees), *Pinus*, *Aesculus*, and several others [149–151]. From the environment, patients inhale basidiospores or desiccated yeasts. Once the propagules reach the lungs, they might develop, multiplicate, and disseminate to other organs, especially to the central nervous system [148,152–154].

The treatment for cryptococcosis is performed with amphotericin B combined with fluconazole and/or 5-flucytosine [148,155–157]. Although resistance is not considered an issue in *Cryptococcus* spp. [157], secondary resistance to azoles has been recurrently reported [149,158–164]. The observations include a study showing that the MIC_50_ and MIC_90_ values of fluconazole have increased 2-fold in a comparison between *C*. *neoformans* isolated in 2017 and strains obtained 10 years earlier in Africa [159], and another that reported that the mean MIC_50_ of fluconazole for clinical cryptococcal isolates increased 2-fold over time, from 4 μg/mL in 2000 to 2012 to 8 μg/mL in 2014 to 2018 [165].

### 5.2 Fungicide-driven resistance: Epidemiological, experimental, and field data

Differently from *A*. *fumigatus* and *Candida* spp., *C*. *gattii* and *C*. *neoformans* do not usually occur in crops, flower beds, and commercial plant-based products. They are found in association with *Eucalyptus* and other trees, especially in trunk hollows [151,166,167]. Thus, it is unusual to link these species with fungicide exposure in the environment, as these chemicals are often employed to preserve and treat plant diseases of commercial relevance [26,28,33]. Nonetheless, it is worth remarking that *Eucalyptus* and other trees are valuable assets for the wood industry, which also uses fungicides for wood preservation [33]. Moreover, Chowdhary and colleagues isolated azole-resistant *A*. *fumigatus* from trunk hollows in Tanzania [75], the same niche of pathogenic *Cryptococcus* [151,166]. On that account, Del Poeta and Casadevall hypothesized that fungicides could also be driving cryptococcus virulence and resistance evolution [168].

Trying to prove this hypothesis, Bastos and colleagues evaluated the effect of the environmental antifungals tebuconazole and pyraclostrobin (a strobilurin that acts as mitochondrial respiration inhibitor) on *C*. *gattii* and *C*. *neoformans* strains. The exposure to agrochemicals caused cross-resistance to medical azoles, remarkably fluconazole. The cross-resistance was permanent in some exposed strains, lasting even after several cultures in agrochemical-free media, and temporary in others, then returning to the original susceptibility when the contact with the fungicide ceased [31,169]. Other studies using a similar methodology and the same strains also demonstrated that exposure to the fungicide benomyl (mitotic inhibitor) and the herbicides flumioxazin (inhibits protoporphyrinogen oxidase, an enzyme that is important for the synthesis of chlorophyll), isoxaflutole (inhibits the 4-hydroxyphenyl pyruvate dioxygenase), and pendimethalin (inhibits root and shoot growth by preventing plant cell division and elongation) reduced the susceptibility to agrochemicals and clinical antifungals (https://www.epa.gov/caddis-vol2/caddis-volume-2-sources-stressors-responses-herbicides) [170,171]. Although herbicides have different mechanisms of action compared to fungicides, they may activate pathways that increase fungal fitness, which probably alter the way that fungal cells behave in the presence of clinical antifungals. Cross-resistance to fluconazole was also verified in an in vivo murine model for cryptococcosis. The drug proved ineffective in controlling the infection caused by cells previously adapted to tebuconazole, pyraclostrobin, and benomyl, compared to cells nonexposed to fungicides [31,169,171].

**Fig 3:**
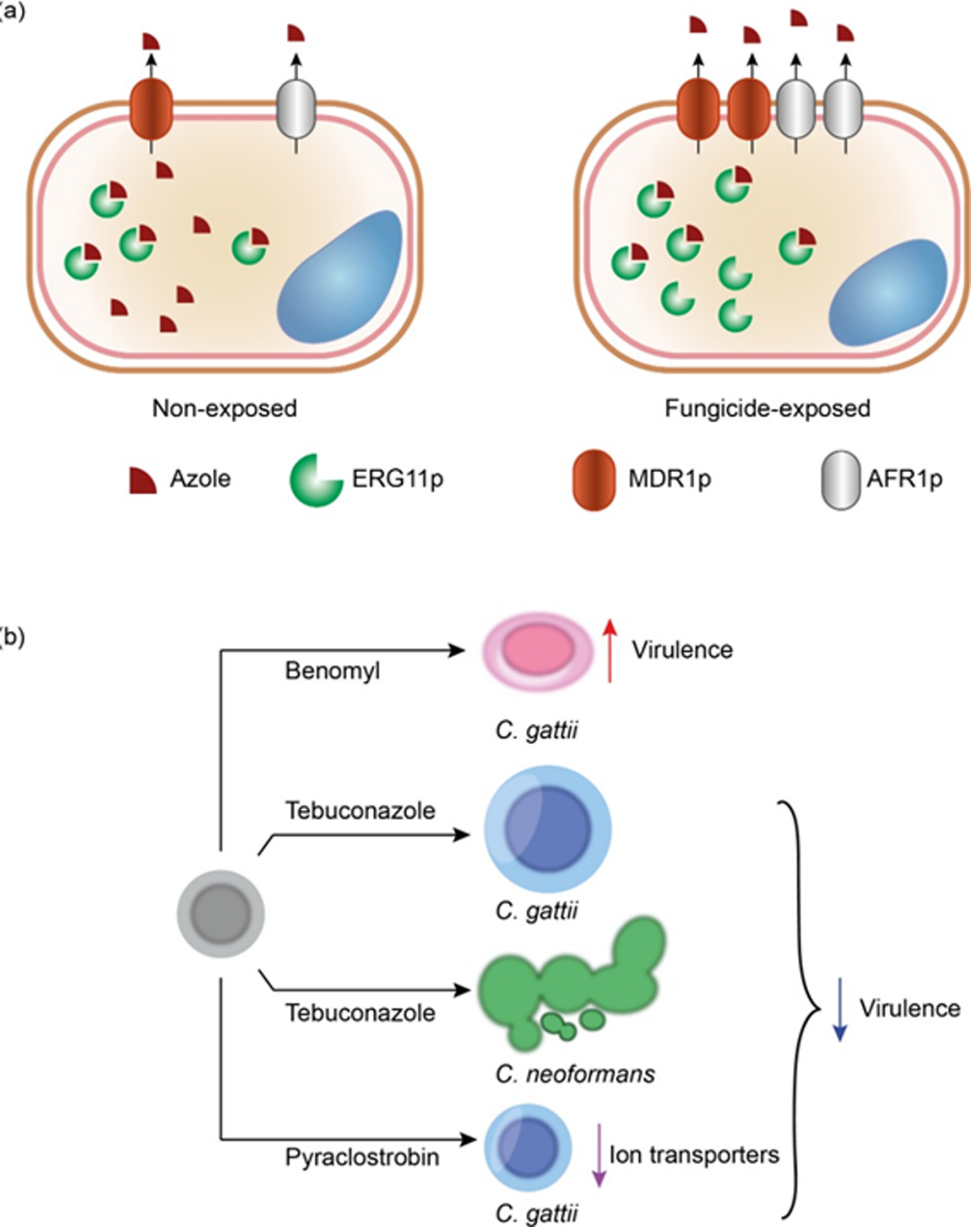
Fungicide exposure effects on *Cryptococcus* spp. (a) Exposure to fungicides can select cross-resistance to clinical azoles especially through overexpression of efflux pumps (*MDR11* and *AFR1*) and *ERG11* genes, the azole target. (b) Different fungicides also can induce important alterations in the cell morphophysiology of *Cryptococcus* cells that may be related to virulence.

*Cryptococcus* spp. usually become more tolerant to azoles through 3 mechanisms: (i) enhanced expression of ERG11p; (ii) mutation in the *ERG11* gene; and (iii) overexpression of efflux pumps [155,172–175]. The molecular mechanism behind cross-resistance selected by environmental azoles, strobilurins, and benzimidazoles, however, has not been fully uncovered. Nonetheless, it seems that fungicide exposure selects mutations in some strains whose resistance strengthens permanently [31,169,171]. It is still unclear the role of mutations in these phenotypes. Epigenetic changes cannot be ruled out since in *C*. *neoformans*, for example, they can remain for a long time in the absence of a stressor agent [176]. On the other hand, the expression analysis of *ERG11* and the efflux pump genes *AFR1*, *PDR11*, and *MDR11* revealed that exposure to tebuconazole, pyraclostrobin, and benomyl boosted their expression in *C*. *gattii* and *C*. *neoformans* (Fig 3A) [31,169,171]. Besides, Carneiro and colleagues performed a rhodamine 6G assay and observed that benomyl-exposed cells pumped out the dye more than the nonexposed control, thus reinforcing this mechanism as a probable factor in the cross-resistance to medical azoles [171].

These data demonstrate that not only DMIs structurally similar to medical azoles select cross-resistance to clinical drugs in *Cryptococcus* spp., but also other fungicides with different targets, and herbicides [31,169–171]. However, a question remains: If fungicide-driven resistance occurs in *Cryptococcus* spp., which are widely spread over natural areas, why has such a small number of azole-resistant *Cryptococcus* been isolated from the environment? The answer may be related to specific conditions that apparently select cross-resistance between fungicides and clinical azoles, such as the temperature [31,169].

The role of temperature in the antifungal tolerance process becomes evident when analyzing cross-resistance. In this case, Bastos and colleagues observed that exposing *C*. *gattii* and *C*. *neoformans* strains to fungicides at 30°C increased the number of colonies that became more resistant to fungicides, compared to when this process was executed at 37°C [31,169]. In addition, the temperature influenced the MIC of azoles used as clinical drugs and fungicides. When the MIC of drugs was determined at 37°C using colonies previously exposed to fungicides at 30°C, the MIC values was lower than when the experiment was carried out at 30°C [31,169]. Another study recently confirmed the connection between resistance acquisition and lower temperature as they proved that adaptation in drugs as fluconazole and amphotericin B at lower temperatures selects resistance to these drugs in *C*. *neoformans*, which does not happen at a higher temperature [177]. Overall, temperature probably influences the survival and adaptation of *Cryptococcus* spp. in the presence of fungicides and clinical drugs, as well as the manifestation of this resistance in a host with high body temperature. It suggests that if the resistance acquisition happens in the environment due to fungicide, *Cryptococcus* may not express it in vivo [31,169].

### 5.3 Fungicide effects on morphology, physiology, and virulence: What we know and it is missing?

It has been proved that fungicides also affect the morphology and virulence of *Cryptococcus* spp [31,169]. As in other fungi, the cell morphology of *Cryptococcus* is crucial to resist environmental stresses and for virulence. Remarkably, the capsule, which is very characteristic of this genus, is deemed as the primary virulence factor [178]. In general, cells with a large capsule tend to be more virulent than those with a small one or acapsular mutants [179]. The surface–volume (S/V) ratio of the yeast is another factor that plays an essential role in the pathogenesis of these species. Yeasts with a high S/V also appear to be more virulent since they replicate fast and migrate to the CNS to a great extent [171,180].

When *C*. *gattii* R265 was exposed to tebuconazole, the cell body expanded (decreased S/V), compared to nonexposed controls (Fig 3B). This phenomenon coincided with a reduced virulence of these cells in the murine model for cryptococcosis, achieving an avirulent status since they were unable to kill any mice [31]. Tebuconazole-exposed *C*. *neoformans* H99 [31] and pyraclostrobin-exposed *C*. *gattii* R265 [169] were also less virulent than non-fungicide-exposed cells, which demonstrated that there is a fitness cost of being more resistant to drugs. In those cases, the decrease in virulence was related to pseudohyphae formation in tebuconazole-adapted *C*. *neoformans* H99 [31] and a reduced expression of ion transporters in pyraclostrobin-exposed *C*. *gattii* (Fig 3B) [169]. Conversely, *C*. *gattii* L24/01, previously nonvirulent, became hypervirulent after exposure to benomyl (Fig 3B). It rapidly translocates to the brain, survives and multiply inside macrophages, and kills mice. This phenotype was associated with the inscrease in the S/V ratio, and an improved replicative capacity, both in vitro and inside phagocytes [171]. Together, these results show how complex could be the fungicide exposure effects on *C*. *neoformans* and *C*. *gattii* morphophysiology and virulence, besides its effect on antifungal resistance.

In summary, these data indicate that fungicide exposure affects the resistance, morphology, and virulence of *Cryptococcus* spp. in a fungicide- and strain-dependent manner. There is also a fitness cost translated as a decrease or loss of virulence in some strains. In contrast, others can become surprisingly more adapted to the host, resulting in a virulence boost.

## 6. Conclusions and perspectives

Several studies have demonstrated that there is an environmental route driving resistance to medical azoles in *A*. *fumigatus* due to fungicide use, especially the use of DMIs. Field and laboratory data revealed that resistant strains found in patients and in the environment could develop cross-resistance to environmental and medical azoles via the same mechanism. Likewise, susceptible isolates can become resistant when exposed to environmental azoles. However, the existing literature is not unanimous on whether or not resistant *A*. *fumigatus* strains hold predominance in azole-contaminated or fungicide-sprayed soils [20,86,102]. One theory rejects the possibility of spontaneous emergence of azole resistance in the soil by suggesting that the phenomenon would be triggered by crop waste gathered up in the surroundings. This hypothesis explains the findings of some authors who observed the prevalence of resistant strains in compost material [20] but not in arable soils [104]. In fact, *A*. *fumigatus* is commonly found in compost piles, plant material in decomposition, and wastewater from urban areas [98]. Thus, studies that did not detect resistant strains eventually assume the soil as hotspot of resistance emergence, when it might actually be importing this condition.

Further studies should clarify why there is an enrichement of resistant isolates in some places containing azoles but not in others. In addition, they should provide a better understanding of the roles of the fungicide application regimen, the accumulation of these substances in the soil, and their influence on resistance development. Other unanswered questions, such as the importance of sexual and parasexual cycles in the process of resistance acquisition, the reasons why TR_34_/L98H and other mutants do not seem to present fitness costs, and how fungicide exposure affects the physiology and virulence of *A*. *fumigatus* strains should also be adressed.

Despite scarce, the existing evidence of an environmental route triggering resistance in pathogenic yeasts (such as *Candida* and *Cryptococcus*) should not be neglected. Most of the current data are based on in vitro studies pointing out that agrochemicals could select cross-resistance to medical azoles. Nonetheless, comprehensive fieldwork comparing the isolation of resistant strains from azole-containing environments versus azole-free ones is still necessary. The studies must also focus on revealing the molecular mechanisms of resistance selected by fungicides and how extrinsic and intrinsic conditions interfere with this phenomenon.

One of the main problems with the environmental drug acquisition is that measures to prevent and control the emergence of resistant strains in clinical practice, including the rational use of drugs, have overall proved to be inefficient, which reinforces the need for new perspectives. The one-health approach has been successful in dealing with antibiotic resistance, as indiscriminate use of these drugs in veterinary medicine and especially as growth promoters for animals has been perceived as a source of acquired bacterial resistance. Thus, there is a great international effort and pressure for the rational use of antibiotics in animal medicine and restriction of their use as growth promoters [181]. In this case, antifungal resistance should also be looked after since the origin of this problem could be in the environment outside the hospital.

Fungicides and other pesticides are indivisible parts of current food production and supply, but assuring human health is paramount, despite productivity claims. Therefore, the sensible use of fungicides with the potential for selecting cross-resistance with clinical drugs is a top priority in future discussions.
